# Combining Intensive Counseling by Frontline Workers with a Nationwide Mass Media Campaign Has Large Differential Impacts on Complementary Feeding Practices but Not on Child Growth: Results of a Cluster-Randomized Program Evaluation in Bangladesh^1^,^2^,^3^

**DOI:** 10.3945/jn.116.232314

**Published:** 2016-08-31

**Authors:** Purnima Menon, Phuong Hong Nguyen, Kuntal Kumar Saha, Adiba Khaled, Tina Sanghvi, Jean Baker, Kaosar Afsana, Raisul Haque, Edward A Frongillo, Marie T Ruel, Rahul Rawat

**Affiliations:** 4 Poverty, Health and Nutrition Division, International Food Policy Research Institute, Washington, DC;; 5 FHI 360, Washington, DC;; 6 BRAC, Dhaka, Bangladesh; and; 7 University of South Carolina, Columbia, SC

**Keywords:** complementary feeding, child undernutrition, cluster randomized trial, effectiveness evaluation, Bangladesh

## Abstract

**Background**: Complementary feeding (CF) contributes to child growth and development, but few CF programs are delivered at scale. Alive & Thrive addressed this in Bangladesh through intensified interpersonal counseling (IPC), mass media (MM), and community mobilization (CM).

**Objective:** The objective was to evaluate the impact of providing IPC + MM + CM (intensive) compared with standard nutrition counseling + less intensive MM + CM (nonintensive) on CF practices and anthropometric measurements.

**Methods:** We used a cluster-randomized, nonblinded evaluation with cross-sectional surveys [*n* = ∼600 and 1090 children 6–23.9 mo and 24–47.9 mo/group, respectively, at baseline (2010) and *n* = ∼500 and 1100 children of the same age, respectively, at endline (2014)]. We derived difference-in-difference impact estimates (DDEs), adjusting for geographic clustering, infant age, sex, differences in baseline characteristics, and differential change in characteristics over time.

**Results:** Groups were similar at baseline. CF improvements were significantly greater in the intensive than in the nonintensive group [DDEs: 16.3, 14.7, 22.0, and 24.6 percentage points (pp) for minimum dietary diversity, minimum meal frequency, minimum acceptable diet, and consumption of iron-rich foods, respectively]. In the intensive group, CF practices were high: 50.4% for minimum acceptable diet, 63.8% for minimum diet diversity, 75.1% for minimum meal frequency, and 78.5% for consumption of iron-rich foods. Timely introduction of foods improved. Significant, nondifferential stunting declines occurred in intensive (6.2 pp) and nonintensive (5.2 pp) groups in children 24–47.9 mo.

**Conclusions:** The intensive program substantially improved CF practices compared with the nonintensive program. Large-scale program delivery was feasible and, with the use of multiple platforms, reached 1.7 million households. Nondifferential impacts on stunting were likely due to rapid positive secular trends in Bangladesh. Accelerating linear growth further could require accompanying interventions. This study establishes proof of concept for large-scale behavior change interventions to improve child feeding. This trial was registered at clinicaltrials.gov as NCT01678716.

## Introduction

Bangladesh, a country of 155 million people with an annual birth cohort of 3.1 million, has made dramatic health advances for its population over the last 2 decades, and is hailed as a remarkable health success story. Although Bangladesh has seen rapid improvements in infant, child, and maternal mortality; immunization coverage; infectious disease treatment coverage; fertility rates; life expectancy; and other indicators of societal development, undernutrition remains a substantial challenge despite recent secular declines (1). In 2014, an estimated 36% of children <5 y of age were stunted, reflecting a 4 percentage point (pp)^8^ decline since 2011, and a 15 pp decline since 2004 (mean rate of decline of 1.5 pp/y). Over the same time period, there has been minimal change in the prevalence of wasting, with an estimated prevalence of 14% in 2014, reflecting a decline of only 1 pp in the last 10 y (2).

Appropriate infant and young child feeding (IYCF) practices, which include exclusive breastfeeding until 6 mo of age and the provision of safe and nutritionally rich foods in sufficient quantity in addition to breastmilk from 6 to 23 mo of age, are a critical component of optimal child growth and development (3–5). In Bangladesh, although the rates of exclusive breastfeeding have increased in the last 10 y to an estimated 55% in 2014, there has been little to no progress in improving the quality of children’s diets, as measured by indicators such as minimum diet diversity and minimum acceptable diet (2). An estimated 26% of children 6–23 mo of age nationally consumed adequately diverse diets, with only 23% consuming a minimally acceptable diet. Traditional complementary foods in Bangladesh, as in many other parts of the developing world, have low energy and micronutrient density and poor protein quality (6–8). Interventions to improve child feeding at scale are imperative because the inadequacy of complementary feeding (CF) in Bangladesh and other similar contexts remains a substantial and somewhat intractable challenge.

Several studies have reviewed the strategies commonly used for improving CF knowledge and behaviors and their impact on child growth, morbidity, and survival (4, 5, 9, 10). Studied interventions have included the provision of nutrition education and/or complementary foods. Pooled results from food-secure and -insecure populations show that education alone increased linear growth by a height-for-age *z* score (HAZ) of 0.23, resulting in a 29% reduction in stunting, as well as a 62% improvement in the uptake of recommended foods (10). In studies conducted in food-insecure settings, complementary food supplements provided with or without education had a larger impact, with an increase of 0.39 HAZ, although stunting rates were not significantly different between the intervention and comparison groups (10). A recent study from Bangladesh reported a small 0.07–0.10 HAZ increase when complementary foods were provided with nutrition education compared with nutrition education alone (11). The reviews and studies available focus primarily on child growth outcomes and generally fail to carefully analyze or report the impacts of CF promotion interventions on learning and adoption of optimal practices by mothers. Moreover, evidence to date is derived predominantly from efficacy studies or small-scale or pilot effectiveness interventions. As a result, evidence is scant on what works to improve maternal knowledge and practices related to CF practices, how these changes in turn lead to positive child outcomes, and what factors enable successful scale-up of these interventions (12).

This paper reports on findings from a cluster-randomized impact evaluation of an at-scale program in Bangladesh. The objectives of our evaluation were to compare the impact of 2 Alive & Thrive (A&T) intervention packages on CF practices and anthropometric outcomes. We were able to find few examples of large scale programs to improve CF practices, and no examples of such programs that were rigorously evaluated. Our study makes a substantial contribution to the literature on improving CF practices through a proof-of-concept rigorous evaluation of a set of interventions delivered at scale.

## Methods

### 

#### Study context and intervention description.

BRAC, a large nongovernmental organization, delivered standard and intensified interpersonal counseling (IPC) and community mobilization (CM) in 50 rural subdistricts in Bangladesh through its existing countrywide essential health care program (13). For standard nutrition counseling, BRAC frontline workers (called Shasthya Kormi) and volunteers [called Shasthya Sebika (SS)] conducted routine home visits and provided information on IYCF practices. In intensive areas, a new cadre of nutrition-focused frontline workers, the Pushti Kormi (PK), together with the SS, conducted multiple age-targeted IYCF-focused counseling visits to households with pregnant women and mothers of children ≤2 y of age, coached mothers as they tried out the practices, and engaged other family members to support the behaviors. The mass media (MM) component, implemented in both intensive and nonintensive areas, consisted of the national broadcast of 7 television spots that targeted mothers, family members, health workers, and local doctors with messages on various aspects of IYCF; 3 of the spots focused on CF. Purchases of media airtime were designed for multiple airings during the country’s most-watched programs. In intensive areas that had low electricity and limited access to television, supplemental activities were conducted to air the television spots and other IYCF films produced by the project through local video screenings. In intensive areas, CM included sensitization of community leaders to IYCF, and community theater shows focused on IYCF. In nonintensive areas, CM was less structured and covered general health care topics such as family planning, pregnancy registration, and antenatal care, and did not include IYCF-related information. Thus, A&T used 3 different platforms, i.e., IPC, CM, and MM, to deliver interventions to targeted beneficiaries. The intensive group received all 3 interventions; the nonintensive group received standard IPC and less-intensive MM and CM.

During the intervention period, A&T facilitated the training of >75,000 frontline workers and health providers across the country. The program model reached a large scale, with an estimated 1.7 million mothers of children <2 y of age in 50 subdistricts accessed by IPC by mid-2014. The MM intervention operated at a national level and through national television channels.

#### Evaluation design.

A cluster-randomized, nonblinded impact evaluation design was used to compare the impact of the 2 A&T intervention packages (NCT01678716). A cross-sectional household survey was conducted at baseline (2010) and exactly 4 y later (2014) in the same communities in households with children 0–47.9 mo of age. We previously reported program impacts on exclusive breastfeeding rates in children 0–5.9 mo of age (14). This paper presents findings on the WHO-recommended core CF practices for children 6–23.9 mo of age and HAZ and stunting in children 24–47.9 mo of age. Children in this age group were assumed to have had the opportunity for program exposure during the critical window of opportunity between pregnancy and 24 mo of age. Although nutrition interventions should reach infants and young children during their first 2 y, the accrued impacts of these interventions ideally should be measured once this period of greatest potential benefit is concluded (15). For the purposes of measuring the impact of interventions on anthropometric measurements, the oldest children (47.9 mo) would have been born just after the baseline survey, at the start of the program implementation, and thus would have had the opportunity to be exposed to the intervention from birth up until they reached 24 mo of age; the youngest children would have been born midway through the program (in 2012) and exposed from birth until 24 mo of age.

#### Sample size estimations.

Sample size calculations were carried out to detect differences in the primary outcomes, i.e., CF practices in children 6–23.9 mo of age and stunting in children 24–47.9 mo of age, between the 2 intervention groups at end line, considering an α of 0.05, a power of 0.80, an intraclass correlation of 0.01 (estimated from previous national or subnational surveys), and an estimated baseline prevalence of the primary outcomes. We hypothesized that the intensive intervention would favor the primary impact indicators; therefore, a 1-sided test was used.

Assuming a baseline prevalence of 44% for minimum dietary diversity, we estimated that a total sample of 980 infants aged 6–23.9 mo (490/group) was sufficient to detect a ≥10-pp difference in the proportion of children achieving minimum dietary diversity at end line. In addition, a total sample of 2020 children aged 24–47.9 mo (1010/group) was sufficient to detect a ≥8-pp difference in the proportion of stunted children at end line, assuming a baseline prevalence of 50%. Before we conducted the end line survey, we reverified our detectable effect sizes on the basis of the original sample size, the observed baseline prevalence values of our primary impact indicators, and the intraclass correlation from the baseline survey. On the basis of these variables, our detectable effect size increased from 10 to 14 pp for minimum dietary diversity, and increased from 8 to 10 pp for stunting.

#### Random assignment and blinding.

One hundred subdistricts across 5 of 6 divisions were selected by BRAC as possible A&T intensive areas on the basis of high poverty and stunting levels and not having been included in the government National Nutrition Program. This list was narrowed to 78 on the basis of geographic proximity, size, and other operational aspects to ensure homogeneity across the sample. Within each division, 4 subdistricts were then randomly selected for inclusion in the evaluation sample with the use of a computer program, for a total of 20 subdistricts. Subdistricts within each division were then randomly assigned with the use of a computer program to either the intensive (10 subdistricts) or nonintensive (10 subdistricts) intervention. The randomization process was carried out in the presence of BRAC and A&T staff and the program evaluators at the BRAC headquarters in Dhaka. Households within the intensive and nonintensive areas were not explicitly made aware of the results of the randomization. In addition, there was no blinding of the intervention at the level of service delivery.

#### Outcomes.

The primary outcomes were CF practices in children 6–23.9 mo of age on the basis of the indicators recommended by the WHO (16), and the prevalence of stunting in children 24–47.9 mo of age. Five CF indicators were examined: *1*) minimum dietary diversity (defined as the consumption by children of foods from ≥4 of 7 food groups in the previous 24 h); *2*) minimum meal frequency as appropriate for age and breastfeeding status; *3*) minimum acceptable diet [defined as breastfeeding and achievement of the minimum dietary diversity (as defined above) in children, as well as age-appropriate minimum meal frequency]; *4*) consumption of iron-rich or iron-fortified food; and *5*) timely introduction of solid, semisolid or soft foods (17). The CF indicators were constructed on the basis of maternal or caregiver previous-day recalls of foods consumed by the target child. Anthropometric data were collected with the use of standard methods (18) and assessments made by trained and standardized field staff. The weight of children was measured with the use of electronic weighing scales precise to 100 g. Locally manufactured collapsible length and height boards, which were precise to 1 mm, were used to measure the recumbent length of children <24 mo of age and the standing height of children ≥24 mo. Weight and length or height were converted into HAZ, weight-for-age *z* score (WAZ), and weight-for-height *z* score (WHZ) according to 2006 WHO child growth standards (19). Stunting, underweight, and wasting were defined as <−2 for HAZ, WAZ, and WHZ, respectively.

#### Statistical analysis.

Baseline differences between the 2 interventions were tested with the use of linear regression models (for continuous variables) or logit regression models (for categorical variables) while accounting for geographic clustering (20). For impact analyses, we derived difference-in-difference impact estimates (DDEs) with the use of fixed-effects regression models that assessed differences between the intensive and nonintensive groups over time (21). We present intention-to-treat DDEs while adjusting for geographic clustering, infant age, and sex, and also a model fully adjusted for geographic clustering, infant age, sex, baseline characteristics that were different between groups, and characteristics that changed differentially over time. Dose–response analyses were conducted with the use of logit regression models while using exposure variables constructed from single and multiple platforms; the different platforms used were IPC, CM, and MM. To confirm the accuracy of self-reported outcome measures, we measured social desirability to assess and account for potential biases in our main impact estimates on maternal-reported CF practices. Social desirability, the tendency of respondents to answer questions or to act in a manner that is viewed favorably by others, was measured with the use of a scale that was based on a subset of 5 items adapted from Reynolds and Gerbasi (22) (**Supplemental Text 1**). Data analysis was performed with the use of STATA 13; a statistical analysis plan was developed before end line data collection and discussed with the funder and program implementers.

#### Ethical approval.

Approval for the study was obtained from the institutional review board at the International Food Policy Research Institute and the Bangladesh Medical Research Council. All mothers of study children were provided with detailed information about the study in writing and verbally at recruitment. Verbal informed consent was obtained from mothers in the survey.

## Results

### 

#### Trial flow and intervention duration.

**FIGURE 1:**
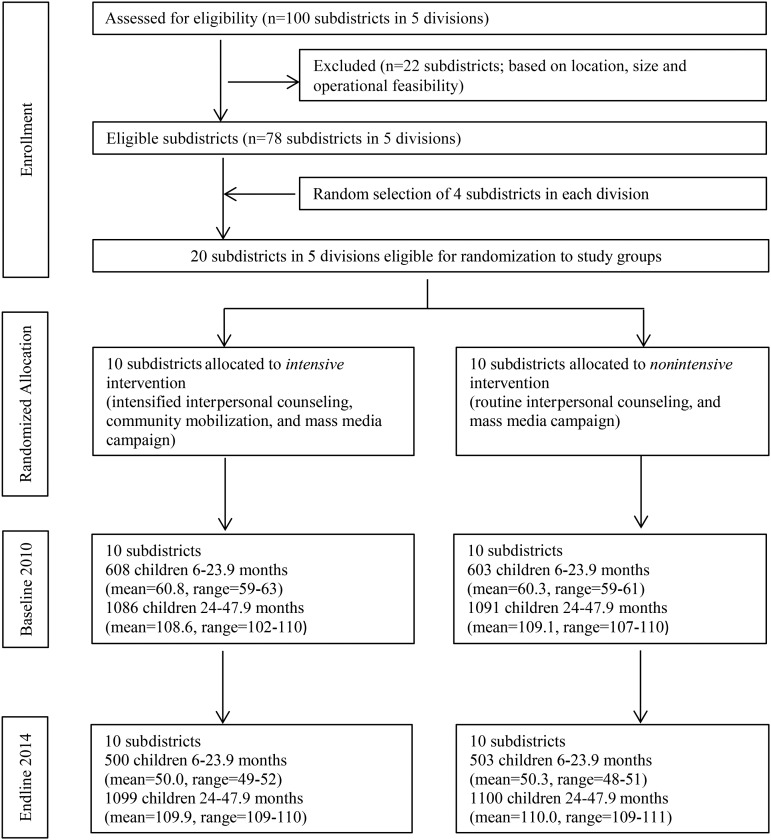
Trial profile.

No evaluation clusters were lost to follow-up (**Figure 1**); none crossed from the nonintensive to the intensive group during implementation. There was little variation in cluster size across clusters and over time.

#### Sample characteristics.

Because children in the 6- to 23.9-mo and 24- to 47.9-mo age groups were sampled separately, we presented baseline characteristics of these 2 groups separately (**Table 1**). The random assignment exercise was successful, and resulted in a well-balanced set of key characteristics that might be related either to intervention uptake or intervention effectiveness. There were no differences at baseline in the core impact indicators of CF practices in children aged 6–23.9 mo and anthropometric outcomes for children 24–47.9 mo of age. In households sampled for an index child aged 6–23.9 mo, we observed significant differences in child anthropometric measurements, women’s occupations, maternal thinness, and household ownership of agricultural land; in households sampled for an index child aged 24–47.9 mo, we observed differences in women’s occupations, maternal thinness, land ownership, maternal age, and the overall household socioeconomic index. We account for these differences at baseline in our fully adjusted impact estimate regression models.

**TABLE 1:** Selected characteristics of the study sample at baseline and end line for children aged 6–23.9 mo and 24–47.9 mo^1^

	Baseline (T1)		End line (T2)				
Characteristics	Intensive	Nonintensive	Intensive	Nonintensive	Intensive T_2_ − T_1_	Nonintensive T_2_ − T_1_	*P*^2^
Children aged 6–23.9 mo, *n*	603	608	500	503			
Household							
Children <5 y of age, *n*	1.22 ± 0.41	1.27 ± 0.44	1.20 ± 0.40	1.23 ± 0.42	−0.02	−0.04	0.61
Ownership of house, %	93.4	95.4	96.2	93.8	2.78*	1.52	0.01
Ownership of garden, %	30.6	30.5	33.8	34.8	3.21	4.28	0.86
Ownership of agriculture land, %	49.7^##^	41.8	53.0^#^	46.0	3.32	4.26	0.72
SES index	−0.03 ± 0.90	0.06 ± 1.01	−0.09 ± 0.78	0.06 ± 1.03	−0.06	−0.01	0.59
Food insecurity, %	32.1	31.0	14.2^##^	22.1	−17.9***	−8.94***	0.22
Maternal factors							
Maternal stress, %	48.9	46.3	27.8^##^	39.8	−21.1***	−6.51*	0.03
BMI, kg/m^2^	19.8 ± 3.07^#^	20.2 ± 2.97	20.6 ± 3.21	20.9 ± 3.39	0.78***	0.70***	0.78
Age, y	26.4 ± 6.06	26.4 ± 5.99	25.9 ± 5.43	25.2 ± 5.43	−0.50	−1.19***	0.15
Schooling, y	4.79 ± 3.57	4.87 ± 3.69	5.67 ± 3.28	5.89 ± 3.35	0.88***	1.02***	0.57
Occupation as housewife, %	96.4^##^	93.0	76.0^#^	81.7	−20.4***	−11.3***	0.10
Maternal dietary diversity, *n*	7.68 ± 1.94	7.88 ± 2.01	8.83 ± 2.12^##^	8.46 ± 1.87	1.15***	0.58***	0.07
Health services access							
Prenatal visit, *n*	2.35 ± 1.92	2.62 ± 1.99	4.35 ± 2.15	3.73 ± 2.03	2.01***	1.10***	0.001
Mothers used iron supplement during pregnancy, %	62.5	63.8	75.4	69.8	12.9***	5.92*	0.16
Child factors							
Sex, %	51.5	49.9	50.8	51.3	−0.68	1.38	0.66
Age, mo	14.9 ± 5.26	14.9 ± 5.20	14.7 ± 4.89	14.5 ± 5.35	−0.27	−0.43	0.76
ARI (2-wk recall), %	58.4	63.7	34.6^###^	46.7	−23.8***	−17.0***	0.15
Diarrhea (2-wk recall), %	14.0	9.78	5.00	4.97	−8.98***	−4.81**	0.12
Children aged 24–47.9 mo, *n*	1086	1091	1099	1100			
Household							
Children <5 y of age, *n*	1.08 ± 0.27	1.09 ± 0.29	1.07 ± 0.25	1.08 ± 0.26	−0.01	−0.01	0.72
Ownership of house, %	95.4	94.8	95.5	93.8	0.01	−0.01	0.48
Ownership of garden, %	28.5	31.5	31.8	34.7	0.03	0.03	0.99
Ownership of agriculture land, %	47.7^###^	39.3	49.3^##^	43.4	1.62	4.04	0.64
SES index	−0.10 ± 0.84	−0.01 ± 0.89	−0.08 ± 0.78	−0.05 ± 0.94	0.02	0.04	0.35
Food insecurity, %	32.6	33.2	16.0^###^	24.2	−16.6***	−8.91***	0.08
Maternal factors							
Maternal stress, %	49.0	48.5	31.0^###^	38.2	−18.0***	−10.3***	0.10
BMI, kg/m^2^	20.5 ± 3.36	20.7 ± 3.23	21.3 ± 3.35	21.5 ± 3.56	0.77***	0.81***	0.78
Age, y	28.1 ± 6.11^#^	27.6 ± 6.01	27.7 ± 6.36^##^	27.0 ± 5.65	−0.61*	−0.40	0.66
Schooling, y	4.46 ± 3.54	4.69 ± 3.64	5.49 ± 3.34	5.35 ± 3.51	1.03***	0.65***	0.07
Occupation as housewife, %	93.4	91.4	69.1^###^	80.6	−24.3***	−10.8***	0.04
Maternal dietary diversity, *n*	7.84 ± 1.92	7.99 ± 1.86	8.70 ± 2.10	8.25 ± 1.99	0.86***	0.26**	0.07
Health services access							
Prenatal visit, *n*	2.16 ± 1.86^#^	2.36 ± 1.93	4.04 ± 2.18^###^	3.42 ± 1.93	1.89***	1.06***	0.001
Mothers used iron supplement during pregnancy, %	59.4	63.2	73.8^#^	69.1	14.4***	5.80*	0.01
Child factors							
Sex, %	54.7	51.4	48.2	50.8	6.47**	0.60	0.03
Age, mo	34.8 ± 6.56	35.2 ± 6.73	35.2 ± 6.83	34.9 ± 6.82	0.49	0.26	0.04
ARI (2-wk recall), %	47.9	52.3	29.2^###^	40.9	−18.7***	−11.4***	0.12
Diarrhea (2-wk recall), %	4.79	7.06	3.82	4.00	−0.97**	−3.0**	0.22

1 Values are means ± SDs or percentages. *,**,***Significant change from baseline to end line: *P < 0.05, **P < 0.01, ***P < 0.001. ^#,##,###^Different from nonintensive at that time: ^#^*P* < 0.05, ^##^*P* < 0.01, ^###^*P* < 0.001. ARI, acute respiratory infection; SES, socioeconomic status; T, time.

2 Significant difference between the changes in intensive compared with nonintensive areas.

#### Impact on reported CF practices.

**FIGURE 2:**
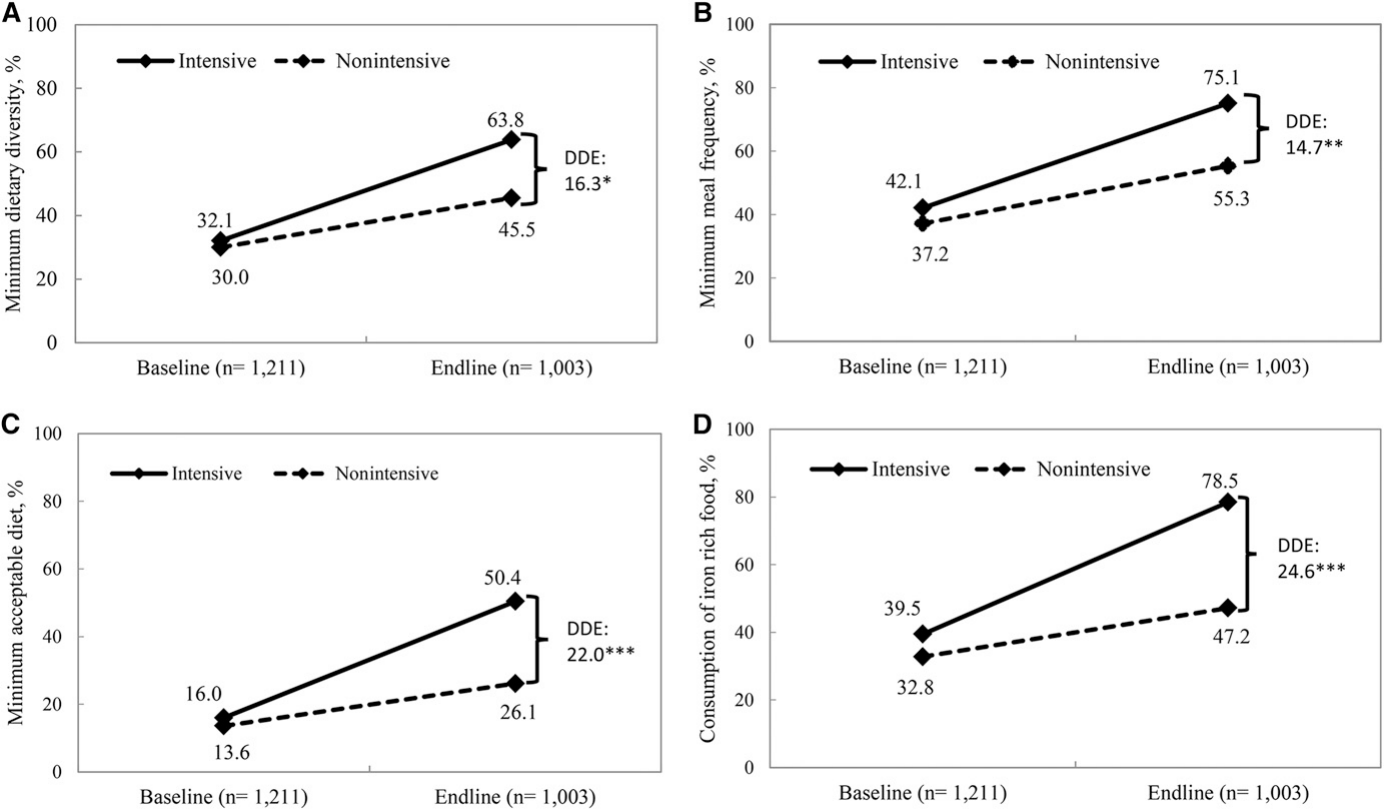
Complementary feeding practices in children aged 6–23.9 mo by program and survey round. Minimum dietary diversity (A), minimum meal frequency (B), minimum acceptable diet (C), and consumption of iron-rich food (D). *,**,***Significantly different: *P < 0.05, **P < 0.01, ***P < 0.001. DDEs with clustered SEs comparing Alive & Thrive intensive and nonintensive areas in 2010 and 2014. Accounts for geographic clustering. DDE, difference-in-difference impact estimate.

The levels of all core WHO CF indicators improved over time (*P* < 0.0001 for all indicators) in both the intensive and nonintensive groups (**Figure 2**). For all CF indicators except timely introduction of solid, semisolid, or soft foods (not shown), the increases were significantly higher in the intensive groups. The DDEs of program impact were 16.3 pp, 14.7 pp, 22.0 pp, and 24.6 pp for minimum dietary diversity, minimum meal frequency, minimum acceptable diet, and consumption of iron-rich foods, respectively. All DDEs were statistically significant in adjusted models. Achieved levels of CF indicators in the intensive areas were high, ranging from 50.4% for minimum acceptable diet to 63.8% for minimum diet diversity, 75.1% for minimum meal frequency, and ≤78.5% for consumption of iron-rich foods.

**FIGURE 3:**
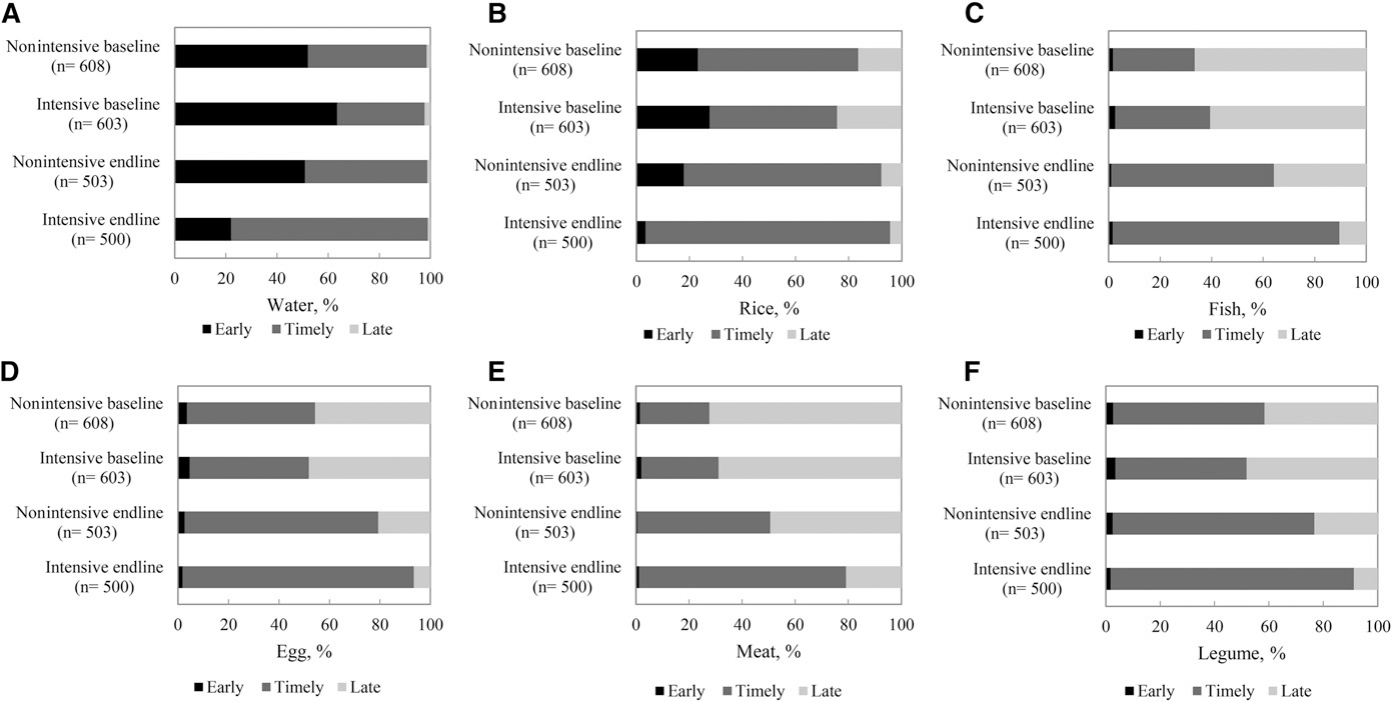
Timely food introduction in children aged 6–23.9 mo, by program and survey round in Bangladesh. Water (A), rice (B), fish (C), eggs (D), meat (E), and legumes (F). Timely introduction of foods was defined as food having been introduced at 6–8.9 mo of age. Differences in difference estimates were 39.2 pp, 28.5 pp, 16.7 pp, 15.6 pp, 22.3 pp, and 20.4 pp for water, rice, fish, eggs, meat, and legumes, respectively. pp, percentage point.

There was also a significant differential shift between groups from early and late introduction of water and other foods to a more well-timed introduction between the ages of 6 and 8.9 mo (**Figure 3**). These program impacts were large and significant, ranging from 16 to 39 pp for different foods. The shift was primarily from early to timely introduction for water, rice, and semisolid foods, and from late to timely introduction for animal-source foods (ASFs) and other foods.

We further assessed the individual food groups consumed by children during the previous 24-h period (**Table 2**). In both the nonintensive and intensive groups, consumption of foods from almost all food groups increased significantly over time. There were statistically significant differential improvements in favor of the intensive group for legumes (14.8 pp), flesh foods (25.4 pp), and eggs (19.1 pp). Given the focus of the program on promoting the consumption of ASFs, we further assessed the impact of the program on different ASFs. We saw similar increases over time within groups for most ASFs, and large program effects on seafood (29.5 pp) and eggs (19.1 pp). These impacts remained significant, with similar magnitudes, in fully adjusted impact models. We also found that household expenditures on these foods were higher in the intervention group households, therefore adding plausibility to our results (results not shown).

**TABLE 2:** Reported intake of foods and food groups in the previous 24 h in children aged 6–23.9 mo by program and survey round in Bangladesh^1^

	Baseline (T1), %		End line (T2), %						
Impact indicators	Intensive *n* = 603	Nonintensive *n* = 608	Intensive *n* = 500	Nonintensive *n* = 503	Intensive T_2_ − T_1_, pp	Nonintensive T_2_ − T_1_, pp	Pure ITT DDE,^2^ pp	Adjusted ITT DDE,^3^ pp	Fully adjusted DDE,^4^ pp
Food group consumption									
Grain	88.2	86.9	97.2	95.6	9.04***	8.73***	0.30	0.00	−0.30
Legumes	21.2	28.0	45.2	37.0	240***	8.95**	15.1**	14.8**	14.0**
Dairy	35.2	44.0	35.0	53.5	−0.20	9.53**	−9.60	−9.60	−10.0
Flesh food	38.7	31.7	77.0	44.1	38.4***	12.5***	25.9***	25.4***	22.5***
Eggs	17.9	19.4	48.4	30.6	30.5***	11.2***	19.3**	19.1**	17.5**
Vegetables and fruits rich in vitamin A	57.6	53.7	68.8	54.9	11.2***	1.14	10.1	9.60	7.70
Other vegetables	25.7	21.2	36.2	27.0	10.5***	5.81*	4.70	4.60	2.00
Animal-source foods									
Meats such as beef, pork, lamb, or goat	3.45	3.65	8.00	10.3	4.55***	6.69***	−2.10	−2.20	−3.20
Chicken, duck, pigeon	4.61	4.81	10.0	6.56	5.39***	1.75	3.70	3.60	2.30
Liver, heart, kidneys or other organ meats	1.32	1.49	7.60	3.38	6.28***	1.89*	4.40*	4.40*	3.80
Fish, prawns, crab, other shellfish, or eels	32.6	24.9	69.4	31.8	36.8***	6.93*	29.9***	29.5***	28.0***
Eggs	17.9	19.4	48.4	30.6	30.5***	11.2***	19.3**	19.1**	17.5**
Sugary and other snack foods									
Chips or Chanachur (spicy, salted snack mix)	20.1	20.1	10.0	13.5	−10.0***	−6.50*	−3.50	−3.80	−3.50
Candies or chocolates	13.2	14.6	8.80	8.55	−4.30*	−6.00*	1.70	1.40	0.20

1 DDEs with clustered SEs compare A&T intensive and nonintensive areas in 2010 and 2014. All *P* values obtained from regression models. *,**,***Significant change from baseline to end line: *P < 0.05, **P < 0.01, ***P < 0.001. A&T, Alive & Thrive; DDE, difference-in-difference impact estimate; ITT, intention to treat; pp, percentage point; T, time.

2 Accounts for geographic clustering only.

3 Accounts for geographic clustering, child sex, and child age.

4 Accounts for geographic clustering, child sex, child age, variables that are different at baseline (mother’s occupation, BMI, and ownership of land), and variables that are different in improvement at baseline and end line (maternal stress, number of prenatal visit, and ownership of house).

We found no evidence of a social desirability bias for any of the CF practices. In both the intensive and nonintensive groups, as social desirability scores increased, there was no commensurate increase in reported CF practices for any indicator (**Supplemental Table 1**).

#### Impact on stunting and other anthropometric indicators.

Stunting declined significantly in children 24–47.9 mo of age in both groups between baseline and end line, by 5.2 pp in the nonintensive and 6.3 pp in the intensive group (**Table 3**). The declines in the prevalence of stunting did not differ between groups, either in the pure intention-to-treat model (*P* = 0.817) or in the fully adjusted model (*P* = 0.784). A similar pattern was observed for the proportion of children classified as being underweight and wasted. In children 6–23.9 mo of age, nondifferential patterns also were observed (Table 3). Improvements in mean HAZ, WAZ, or WHZ did not differ between groups for either age group of children (Table 3 and **Supplemental Figure 1**).

**TABLE 3:** Anthropometric indicators in children aged 6–23.9 mo and 24–47.9 mo by program and survey round^1^

	Baseline (T1)		End line (T2)						
	Intensive	Nonintensive	Intensive	Nonintensive	Intensive T_2_ − T_1_	Nonintensive T_2_ − T_1_	Pure ITT DDE^2^	Adjusted ITT DDE^3^	Fully adjusted DDE^4^
Children 24–47.9 mo of age, *n*	1086	1091	1099	1100					
Stunting	51.8	50.0	45.5	44.8	−6.22**	−5.20*	−1.00	−1.10	−1.60
HAZ	−2.08 ± 1.12	−2.00 ± 1.18	−1.86 ± 1.17	−1.86 ± 1.24	0.22***	0.14**	0.07	0.07	0.02
Underweight	48.3	44.4	41.7	39.3	−6.58**	−5.17*	−1.40	−1.50	−1.80
WAZ	−1.97 ± 1.02	−1.88 ± 1.01	−1.76 ± 1.00	−1.73 ± 1.05	0.21***	0.16***	0.05	0.05	−0.01
Wasting	19.2	17.9	16.7	16.4	−2.53	−1.41	−1.10	−0.70	−1.00
WHZ	−1.16 ± 1.10	−1.07 ± 1.11	−1.03 ± 1.05	−0.98 ± 1.09	0.13**	0.08	0.05	0.04	0.01
Children 6–23.9 mo of age, *n*	603	608	500	503					
Stunting	45.4^###^	35.2	38.1	32.5	−7.32*	−2.72	−4.60	−4.70	−4.60
HAZ	−1.87 ± 1.32^###^	−1.54 ± 1.38	−1.53 ± 1.43	−1.36 ± 1.45	0.34***	0.18*	0.17	0.17	0.20
Underweight	43.4^###^	33.8	34.3	29.3	−9.20**	−4.50	−4.80	−4.70	−4.50
WAZ	−1.75 ± 1.21^##^	−1.58 ± 1.08	−1.59 ± 1.09	−1.39 ± 1.12	0.16*	0.19**	−0.03	−0.02	−0.00
Wasting	21.6	18.2	20.2	17.7	−1.33	−0.51	−0.80	−0.80	−0.90
WHZ	−1.08 ± 1.24	−1.06 ± 1.11	−1.11 ± 1.19	−0.93 ± 1.26	−0.03	0.12	−0.15	−0.15	−0.21

1 Values are means ± SDs or percentages. DDEs with clustered SEs compare A&T intensive and nonintensive areas in 2010 and 2014. All *P* values obtained from regression models. *,**,***Significant change from baseline to end line: *P < 0.05, **P < 0.01, ***P < 0.001. ^#,##,###^Different from nonintensive at that time: ^#^*P* < 0.05, ^##^*P* < 0.01, ^###^*P* < 0.001. A&T, Alive & Thrive; DDE, difference-in-difference impact estimate; HAZ, height-for-age *z* score; ITT, intention to treat; T, time; WAZ, weight-for-age *z* score; WHZ, weight-for-height *z* score.

2 Accounts for geographic clustering only.

3 Accounts for geographic clustering, child sex, and child age.

4 Accounts for geographic clustering, child sex, child age, variables that are different at baseline (mother’s age, ownership of land, and number of prenatal visit), and variables that are different in improvement at baseline and end line (occupation, number of prenatal care, iron folic supplement, child age and sex).

#### Intervention exposure.

In the 6 mo preceding the end line survey, there was significantly greater exposure to a BRAC PK and SS trained to deliver nutrition-related IPC as part of A&T in the intensive group than in the nonintensive group (**Table 4**). Exposure to A&T-supported MM ranged from 34% to 70% for different television spots and was higher in the intensive group for all television spots. Exposure to any CM session was 41% in intensive areas, and absent in nonintensive areas. We constructed exposure categories to capture all 3 categories of interventions (IPC, MM, and CM) and combinations of these; exposure to the full package of interventions was >3 times greater in the intensive than in the nonintensive group.

**TABLE 4:** Exposure to IPC, MM, and CM in children 6–23.9 mo of age^1^

	Intensive (*n* = 500)	Nonintensive (*n* = 503)	Total (*n* = 1003)
Exposure to IPC during the previous 6 mo			
Visited by PK	92.0		45.9
Visited by SS	89.0	15.5	52.1
Exposure to MM			
Ever watched TVC 3	51.4^###^	34.0	42.7
Ever watched TVC 4	69.6^##^	60.4	65.0
Ever watched TVC 5	61.4^###^	49.3	55.3
Ever watched TVC 6	61.6^###^	47.5	54.5
Exposure to any TVC	73.4^#^	67.2	70.3
CM			
Ever attended theater	15.4	0.60	7.98
Ever watched television show	36.6	0.60	18.5
Exposure to any CM	41.2	0.00	20.5
Multiple platform exposure			
No exposure	1.39	28.3	14.9
Exposure to MM alone	2.39	56.1	29.4
Exposure to IPC alone	21.9	4.35	13.1
Exposure to CM alone	0.00		0.00
Exposure to MM + IPC	33.1	11.3	22.1
Exposure to MM + CM	0.80		0.40
Exposure to IPC + CM	3.39		1.69
Exposure to MM + IPC + CM	37.1		18.5

1 Values are percentages. Data are from end line survey. ^#,##,###^Different from nonintensive at that time: ^#^*P* < 0.05, ^##^*P* < 0.01, ^###^*P* < 0.001. CM, community mobilization; IPC, intensified interpersonal counseling; MM, mass media; PK, Pushti Kormi; SS, Shasthya Sebika; TVC, television commercial.

#### Dose–response analyses.

We observe a strong dose–response association between exposures to >1 platform and improved knowledge and feeding practices (**Table 5**). For all 4 CF practices, exposure to MM alone was not significantly associated with improved practices compared with no exposure. Exposure to IPC alone was significantly associated with a 2- to 3-fold higher odds of improved CF practices, with the exception of the achievement of minimum dietary diversity. Exposure to IPC + MM was associated with a 1.7–3.5 fold greater odds of improved CF practices. Exposure to the greatest number of intervention platforms, i.e., to IPC + MM + CM, was associated with increased odds of improved CF practices ranging from 2.8–5.9 fold greater odds for different CF practices compared with no exposure. There was no similar discernible pattern of exposure to combinations of program interventions with stunting or HAZ, although exposure to MM or IPC alone was associated with lower odds of stunting.

**TABLE 5:** Association between exposure to multiple intervention platforms and complementary feeding practices in children aged 6–23.9 mo and 24–47.9 mo^1^

Intervention platform	Minimum dietary diversity^2^ (*n* = 1003)	Minimum meal frequency (*n* = 1003)	Minimum acceptable diet (*n* = 1003)	Consumption of iron-rich food (*n* = 1003)	CF knowledge (*n* = 1003)	Stunting^3^ (*n* = 2199)	HAZ (*n* = 2199)
No exposure	Ref (42.7%)^4^	Ref (51.3%)	Ref (24.0%)	Ref (42.7%)	Ref [2.79]	Ref (54.1%)	Ref [−2.03]^5^
Exposure to MM alone	1.12 (0.73, 1.70)	1.31 (0.87, 1.98)	1.04 (0.65, 1.67)	1.31 (0.87, 1.99)	0.29 [0.14, 0.44]***	0.73 (0.59, 0.97)*	0.077 [−0.06, 0.21]
Exposure to IPC alone	1.43 (0.89, 2.29)	2.31 (1.42, 3.77)**	2.05 (1.23, 3.41)**	2.84 (1.75, 4.62)***	0.57 [0.38, 0.72]***	0.68 (0.49, 0.94)*	0.099 [−0.07, 0.27]
Exposure to MM + IPC	1.74 (1.12, 2.72)*	2.14 (1.37, 3.36)**	2.39 (1.48, 3.85)***	3.51 (2.22, 5.57)***	0.59 [0.42, 0.73]***	0.83 (0.60, 1.12)	0.070 [−0.09, 0.23]
Exposure to MM + IPC + CM	2.78 (1.75, 4.42)***	3.62 (2.22, 5.93)***	3.82 (2.34, 6.22)***	5.93 (3.58, 9.82)***	0.83 [0.65, 0.98]***	1.02 (0.75, 1.41)	−0.15 [−0.32, 0.01]

1 Values are ORs (95% CIs) for mimimum dietary diversity, minimum meal frequency, minimum acceptable diet, consumption of iron-rich food and stunting. Values are βs [95% CIs] for CF knowledge and HAZ. Intensive and nonintensive groups combined. Data are from end line survey. Model adjusted for maternal characteristics (age, education, occupation), child characteristics (sex, birth weight, ARI, diarrhea) and household characteristics (number of children <5 y of age, SES and food security). *,**,***Significantly different: *P < 0.05, **P < 0.01, ***P < 0.001. ARI, acute respiratory infection; CF, complementary feeding; CM, community mobilization; HAZ, height-for-age *z* score; IPC, intensified interpersonal counseling; MM, mass media; Ref, reference.

2 Infant and young child feeding indicators for children 6–23.9 mo of age.

3 Anthropometric indicators for children 24–47.9 mo of age. Model adjusted for maternal characteristics (age, education, occupation, and height), child characteristics (age, age squared, sex, birth weight, ARI, and diarrhea), and household characteristics (number of children <5 y of age, socioeconomic status, and food security).

4 Prevalance of complementary feeding for reference group.

5 Mean HAZ for reference group.

## Discussion

A program providing intensified IPC, MM, and CM (the A&T intensive intervention) at scale had a substantial and significant impact on several CF practices in comparison with changes observed with a less intensive behavior change intervention in Bangladesh. Although improvements in child growth were observed in both groups and for all age groups over time, the DDEs for linear growth and stunting at 24–47.9 mo were not statistically significant; hence, we cannot attribute improvements to the A&T intensified interventions. In contrast to previous studies that have examined impacts on feeding behavior and/or growth in relatively controlled settings (efficacy trials), this study documents significant improvements in a large-scale program. The program was delivered over 4 y, reaching nearly 2 million Bangladeshi families in 50 subdistricts. The MM intervention was delivered nationwide via national television channels; the IPC and CM interventions in the intensive areas first were implemented in 50 of 493 rural subdistricts, and later integrated with BRAC’s other health service platforms. Furthermore, the program engaged with the national government’s program by forming strategic partnerships with the Institute of Public Health Nutrition under the Ministry of Health and Family Welfare.

This study addressed 3 key questions. *1*) Compared with the nonintensive program, did the A&T intensive program improve CF practices? *2*) Were there differential improvements in child anthropometric indicators? *3*) What supports scale-up? We discuss each of these 3 questions below.

### Does the intervention work to improve practices?

Our findings indicate significant and large impacts on practices related to CF. The plausibility of the impacts on CF practices are supported by our findings on the reach of the interventions, which showed that interventions were delivered with quality (23) and reached communities at scale and with good intensity. They also are supported by a dose–response analysis that shows that greater exposure was associated with better practices. The plausibility of findings is also supported by greater reported consumption of specific foods promoted by the program, such as eggs, fish, and vegetables, as well as lower reported consumption of specific foods explicitly identified as nondesirable foods, such as cookies and snacks. Finally, these findings at the child-level reported that intake is in line with household expenditure data and household dietary diversity data, thus providing triangulation across the data collected on factors that might lead to increased child-level diet diversity. Gains in timely introduction of different foods came from the delayed introduction of water and semisolid gruels (from 3–4 mo of age at baseline to >6 mo of age at end line in the intensive areas), as well as from the timely introduction of ASFs (e.g., from ≥8–9 mo for eggs, fish, and liver at baseline to 6–8 mo of age for these foods at end line).

### Do anthropometric indicators improve differentially?

Our findings indicate that a differential change in anthropometric measurements was not seen between the intervention areas. This lack of a differential change in anthropometric outcomes between intensive and nonintensive areas over time could be explained by *1*) the overall trend in improvement in anthropometric outcomes in Bangladesh, *2*) constraints on linear growth not addressed by the interventions, *3*) the inability to adhere completely to CF recommendations on a routine basis, or *4*) illness and/or poorer growth trajectories because of low birth weight and other factors not measured in this evaluation.

First, the trend in improvement in the nutritional status of children in Bangladesh is fairly strong (1.5 pp/y) (2). A difference between the intensive and nonintensive areas therefore would require an additional differential acceleration of this trend, which likely is challenging without a more extensive set of interventions.

Second, the intensive interventions tested in this program focused exclusively on behavior change with the use of multiple platforms to delivery CF education and support. No additional interventions were provided, which could have addressed other causes of constrained linear growth (3). In food-secure populations, it is estimated that CF education alone could lead to a modest improvement on height of 0.23 SD, and no impact on stunting (10). In food-insecure populations, to our knowledge, only one study from Bangladesh on nutrition education alone demonstrated a statistically significant impact on HAZ and a reduction in the risk of stunting (24). Another recent study, which combined behavior-change communication interventions along with food supplements, found small but significant impacts on linear growth and on stunting (11). In our study population, about one-third of households were food insecure (25). Qualitative research conducted as part of a midstudy process evaluation (26) indicated that food availability and resources did constrain routine and sustained practice of some recommendations, especially the purchase and consumption of ASFs (23).

Third, although there were differential improvements in several CF practices, at end line, only one-half of all children consumed what is considered to be a minimally acceptable diet and less than three-quarters consumed a minimally diverse diet. Therefore, the levels achieved may not have been sufficient to translate into improved growth.

Fourth, more than one-half of children reported ≥1 common childhood illness at baseline, reflective of an overall high pattern of child morbidity in this population, which might have contributed to limited impacts on linear growth.

### What lessons does this example offer for scaling up?

Lessons learned for programming to improve CF practices at scale are worth noting in the context of this program evaluation. First, the behavior change interventions tested were developed through formative research for program design; their roll-out and scale up were done through monitoring of quality and coverage; the intervention delivery also included several supervision and management approaches that supported implementation (12, 27). From an implementation science perspective, the approaches used in delivering this set of interventions offer several lessons beyond those available from smaller-scale efficacy trials of behavior change interventions (13). The most critical lessons for scaling up relate to an explicit focus on specific behavior change goals; ensuring adequate investments in intervention design; using data to make decisions about coverage and intervention quality; and ensuring the availability of adequate, stable, and flexible financing for delivery (12). At the same time, the evaluation results suggest that programs that aim to improve CF practices in food-insecure and poor environments should carefully consider complementing the behavior change interventions with complementary interventions that can help address financial and resource constraints to adoption of optimal practices. Programs that aim to achieve an impact on child nutritional status will also need to consider how to address the other determinants of child growth, such as maternal nutrition, sanitation, and poverty. This could be done by incorporating other tailored interventions to specifically address other constraints.

Some limitations of our program evaluation include the following. First, the evaluation areas comprised a smaller geographic area than the total coverage of the program; in a midline evaluation, however, we randomly assessed service delivery in a subset of intensive areas not included in the impact evaluation areas and found no evidence of differential intervention coverage, exposure, or household practices, suggesting limited evaluation bias. Second, in this large-scale programmatic setting, for practical considerations, a cross-sectional evaluation design that sampled children on the basis of the potential of having been exposed to, and benefited from, the intervention was used, rather than tracking individual children. This design precluded our ability to fully link individual child-level exposure to program interventions to growth outcomes for the same children. Third, the intervention was not blinded to implementers or community members, and measurement of CF practices was based on maternal recall. These factors could lead to social desirability in reporting. We found no intervention-specific differentials in socially desirable reporting for CF practices, however (22). Reported dietary diversity also increased with child age in both study arms, as expected, adding plausibility to the measure. Fourth, we did not have the ability to assess the impact of MM alone, because the media campaign was implemented nationwide. We also were unable to isolate the impact of the additional nutrition worker, the PK, without a control area that did not have any BRAC worker. The impact estimates thus may have underestimated the full potential of such a multipronged intervention.

In conclusion, CF is recognized as an important contributor to poor child nutrition and child development globally, but progress is held back (28) because of limited experience with large-scale program strategies that integrate a necessary set of evidence-based interventions (29). With a cluster-randomized evaluation design, we demonstrated that multiplatform behavior change interventions reach households with both scale and intensity, and that this approach improves several CF practices. The intensive program, and its impact on CF practices, however, may not have been enough to differentially accelerate the positive and rapid improvements in linear growth and stunting observed in the target population at the time of the study.

As the global momentum for investing in nutrition ramps up, there is an urgent need for demonstrated large-scale solutions for improving the most fundamental of nutrition actions, i.e., home-based behaviors to improve the quality of diets. This study offers compelling evidence that such interventions can be implemented at scale to deliver impact on what remains a substantial global challenge—improving children’s diets.

## Footnotes

8 Abbreviations used: A&T, Alive & Thrive; ASF, animal-source food; CF, complementary feeding; CM, community mobilization; DDE, difference-in-difference impact estimate; HAZ, height-for-age *z* score; IPC, intensified interpersonal counseling; IYCF, infant and young child feeding; MM, mass media; PK, Pushti Kormi; pp, percentage point; SS, Shasthya Sebika; WAZ, weight-for-age *z* score; WHZ, weight-for-height *z* score.
